# Comparative effectiveness of nature-based social prescribing to alleviate loneliness in Spain: a decision analysis informed by the RECETAS trial

**DOI:** 10.3389/fpsyg.2026.1891830

**Published:** 2026-10-08

**Authors:** Veronika Papon, Beate Jahn, Gaby Sroczynski, Jill S. Litt, Laura Coll-Planas, Montse Masó-Aguado, Marjan Arvandi, Júlia Santamaria Navarro, Igor Kuchin, Sibylle Puntscher, Uwe Siebert, Ursula Rochau

**Affiliations:** 1 Institute of Public Health, Medical Decision Making and Health Technology Assessment, Department of Public Health, Health Services Research and Health Technology Assessment, UMIT TIROL - University for Health Sciences and Technology, Hall in Tirol, Austria; 2 Institute for Global Health (ISGlobal), Barcelona Biomedical Research Park (PRBB), Barcelona, Spain; 3 CIBER Epidemiología y Salud Pública (CIBERESP), Madrid, Spain; 4 Universitat Pompeu Fabra (UPF), Barcelona, Spain; 5 Research Group On Methodology, Methods, Models and Outcomes of Health and Social Sciences (M3O), Faculty of Health Sciences and Welfare, Centre for Health and Social Care Research (CESS), University of Vic-Central University of Catalonia (UVic-UCC), Barcelona, Spain; 6 Institute for Research and Innovation in Life Sciences and Health in Central Catalonia (IRIS-CC), Vic, Spain; 7 Center for Health Decision Science, Departments of Epidemiology and Health Policy & Management, Harvard Chan School of Public Health, Boston, MA, United States; 8 Institute for Technology Assessment and Department of Radiology, Massachusetts General Hospital, Harvard Medical School, Boston, MA, United States

**Keywords:** decision-analytic model, health-related quality of life, loneliness, Markov state-transition model, nature-based social prescribing, QALY, RECETAS

## Abstract

**Background:**

Social prescribing appears to be an effective intervention to reduce loneliness. More evidence is needed on the long-term benefits of nature-based social prescribing (NBSP) to alleviate loneliness.

**Objective:**

We aimed to evaluate the long-term effectiveness, in terms of life expectancy (in life years: LY), quality-adjusted life expectancy (in quality-adjusted life years: QALYs), and loneliness-free years (LFYs), of a NBSP intervention compared to active control (AC) as loneliness-reduction strategy among adults in Spain.

**Methods:**

We developed a Markov state-transition model where participants moved between different health states to estimate LYs, QALYs, and LFYs. Loneliness, reduced loneliness, or relapsed loneliness, was combined with either being in good health or one of the following loneliness-related diseases: cardiovascular diseases, diabetes mellitus type2, depression, anxiety, and dementia. Death from other causes, loneliness-disease-related death and suicide were considered. A lifelong time horizon was applied to evaluate long-term health outcomes of NBSP and AC. The model was populated with data from the “Friends in Nature” (FiN) pragmatic randomized controlled trial conducted in Barcelona (Spain) within the EU Horizon 2020 RECETAS project supplemented with Spanish data. FiN, a nature-based, facilitator-led group-based social prescribing model was compared to AC. We followed a Spanish cohort with a mean age of 61 years, comprising 80% women, experiencing loneliness. Several scenario and sensitivity analyses were performed.

**Results:**

NBSP resulted in 23.98 LYs, 15.15 QALYs, and 10.73 LFYs, versus 23.68 LYs, 14.76 QALYs, and 8.11 LFYs with AC. This corresponds to gains of 0.30 LYs, 0.39 QALYs and 2.62 LFYs for a Spanish cohort of lonely adults. Results remained robust in scenario analyses. Deterministic sensitivity analysis showed that all incremental outcomes were most sensitive to assumptions regarding the relative intervention effectiveness in reducing loneliness after six to twelve months, the relapse probability during this period, and the increased mortality risk associated with loneliness. Results of the PSA suggested NBSP as the optimal strategy under parameter uncertainty in terms of LYs, QALYs, and LFYs in 89% of all simulations.

**Conclusion:**

Our analyses suggest that NBSP is effective over the long-term in reducing loneliness and improving health outcomes in Spain. Further research, including a cost-effectiveness analysis, is needed to better inform decision-makers and support the implementation of NBSP within healthcare systems across different countries.

**Clinical trial registration:**

ClinicalTrials.gov, identifier NCT05488496.

## Introduction

Loneliness, according to a recent report from the World Health Organization (WHO), is a public health concern (World Health Organization, 2025). The absence of loneliness is essential for social health, which, alongside mental and physical health, constitutes a state of complete health, rather than merely the absence of disease (World Health Organization, 2025). Loneliness is a subjective negative phenomenon resulting from a discrepancy between preferred and actual social relationships (Perlman and Peplau, 1981). The prevalence of loneliness is high around the world, ranging from 9% in south-east Asia, 11% in western Europe, 14% in the eastern mediterranean region, and more than 27% in eastern Europe (Surkalim et al., 2022). In Spain, an estimated 13% of the population experiences loneliness (Jiménez Rodríguez, 2024; Fundación ONCE and Fundación AXA, 2024). Among others, depression, anxiety, diabetes mellitus type 2 (T2DM), and cardiovascular diseases (CVD) are associated with loneliness (World Health Organization, 2021; Hackett et al., 2020; Mann et al., 2022; Valtorta et al., 2016; Wang et al., 2023). It also reduces quality of life and increases the risk of suicide attempts and death by suicide (World Health Organization, 2021; Chang et al., 2017; Fässberg et al., 2012; Stickley and Koyanagi, 2016; Vespa et al., 2023; Zheng et al., 2026). Potential mechanisms are related to psychological and biological changes as well as lifestyle factors (Baarck and Kovacic, 2022; Holt-Lunstad and Smith, 2016). A loneliness feedback loop, together with impaired self-regulation and reduced sleep quality, lead to adverse outcomes on health (Hawkley and Cacioppo, 2010). At the physiological level, genetic and neuroendocrine dysregulation lead to higher blood pressure, increased inflammatory activity, and worse immune function (Hawkley and Cacioppo, 2010).

Various interventions have been evaluated to reduce loneliness (Masi et al., 2011; Lasgaard et al., 2026; Patil and Braun, 2024). Early classifications of intervention types focused on social skills, social support, social interaction, or social cognitive training (Masi et al., 2011), while more recent frameworks extended these categories to social support, social network, social and emotional skills, psychological, and psychoeducation (Lasgaard et al., 2026). Similarly, Gardiner et al. (2018) categorized loneliness interventions thematically into social facilitation, psychological approaches, health and social care interventions, animal-assisted interventions, friendship programs and skill-building activities. Overall, a common way to alleviate loneliness across these approaches is to (re)connect people with their social environment and community. This can be also achieved through Social Prescribing (SP). SP aims to identify “a person with non-medical, health-related social needs” (Muhl et al., 2023). The identifier, usually a clinical professional, refers people to “non-clinical supports and services within the community” (Muhl et al., 2023), such as housing subsidies or arts activities (e.g., painting) (Leavell et al., 2019; Chatterjee et al., 2017). Nature-based social prescribing (NBSP) adds a nature component to SP and is a tool to target loneliness by connecting people within nature-based group activities, such as gardening, nature walks, cultural heritage experiences, and bird-watching (Leavell et al., 2019; Litt et al., 2024). Group activities in nature, especially conducted over a longer time, were shown to be effective to reduce loneliness (Lavelle Sachs et al., 2024). However, the evidence base is limited by a lack of large-scale trials and long-term follow-up studies. To our knowledge, particularly, only few high-quality studies, such as randomized controlled trials (RCTs) are available so far (Coventry et al., 2021; Robinson and Breed, 2019; Husk et al., 2020).

The RECETAS consortium (Re-imagining Environments for Connection and Engagement: Testing Actions for Social Prescribing in Natural Spaces) investigates the impact of an NBSP intervention, called “Friends in Nature” (FiN), on loneliness and health-related quality of life (HRQoL) in three pragmatic randomized controlled clinical trials (RCT) and three pre-post observational studies (Litt et al., 2024; Coll-Planas et al., 2024; Santos-Tapia et al., 2023). In the RCT conducted in Barcelona, Spain, 320 participants were randomized 1:1 to either FiN or an active control (AC) (Coll-Planas et al., 2024). FiN consisted of an in-depth motivational interview plus nine two-hour weekly organized activities and participants were followed over a maintenance phase of one year from baseline. In the AC arm, participants received information in a personal interview as sign-posting NBSP about locally available nature-based activities but were not offered further organizational support. The target group included adults from low socio-economic urban areas. The primary outcomes, change in loneliness and HRQoL, were assessed at baseline, three, six, and twelve months follow-up (Coll-Planas et al., 2024).

However, evidence on the long-term effects of NBSP interventions is sparse, as such evidence would require extended follow-up periods in clinical trials. Decision-analytic modeling addresses this gap by integrating short-term and long-term evidence, enabling analysis over a longer time horizon to support decision-making in public health by systematically evaluating expected outcomes of different strategies (Siebert, 2003; Hunink et al., 2014). We consider three complementary outcomes: life years (LYs), quality-adjusted life years (QALYs), and loneliness-free years (LFYs). LYs estimate survival, while QALYs combine length and quality of life and enable comparison across interventions (Drummond et al., 2015). LYs and QALYs are standard in health economic evaluations and are recommended for Spain (Drummond and McGuire, 2001; López-Bastida et al., 2010). However, both may lack sensitivity to changes in specific social outcomes, such as loneliness. To address this, we include LFYs, defined as time with feelings of reduced loneliness. LFYs provide a more directly interpretable measure of intervention effects on loneliness and may be particularly relevant for FiN, where reducing loneliness is a primary goal.

A recent systematic literature review reported only two published models for interventions targeting loneliness (Rochau et al., 2025). Neither model evaluated NBSP interventions, and both were restricted to a five-year time horizon (McDaid and Park, 2021; Engel et al., 2021).

The aim of our study was to develop, validate, and apply a decision-analytic model (DAM) informed by the FiN trial in Barcelona, Spain, to evaluate the long-term health outcomes, measured in LYs gained, QALYs, and LFYs of an NBSP intervention compared to AC over a lifetime horizon in lonely adults in Spain.

## Methods

### Model design

We developed, validated and applied a DAM to assess the impact of a NBSP strategy based on FiN, compared to AC, for alleviating loneliness in Spain (Siebert, 2003). The model was developed in accordance with national Spanish guidelines (López-Bastida et al., 2010), ISPOR-SMDM Modeling Good Research Practices (Siebert et al., 2012; Roberts et al., 2012; Eddy et al., 2012; Caro et al., 2012; Briggs et al., 2012), and is reported following the Consolidated Health Economic Evaluation Reporting Standards (CHEERS) (Husereau et al., 2022). A Markov state-transition model was applied to extrapolate FiN outcomes from the twelve-month RCT in Barcelona, Spain, to a lifetime horizon, integrating additional data sources representative of the lonely adult population in Spain (Siebert, 2003; Siebert et al., 2012; Caro et al., 2012). Given the manageable number of health states, defined by combinations of loneliness and diseases, we used a cohort simulation (Siebert et al., 2012; Caro et al., 2012). The model allows for transitions between mutually exclusive health states over a lifetime (Figure 1).

**Figure 1:**
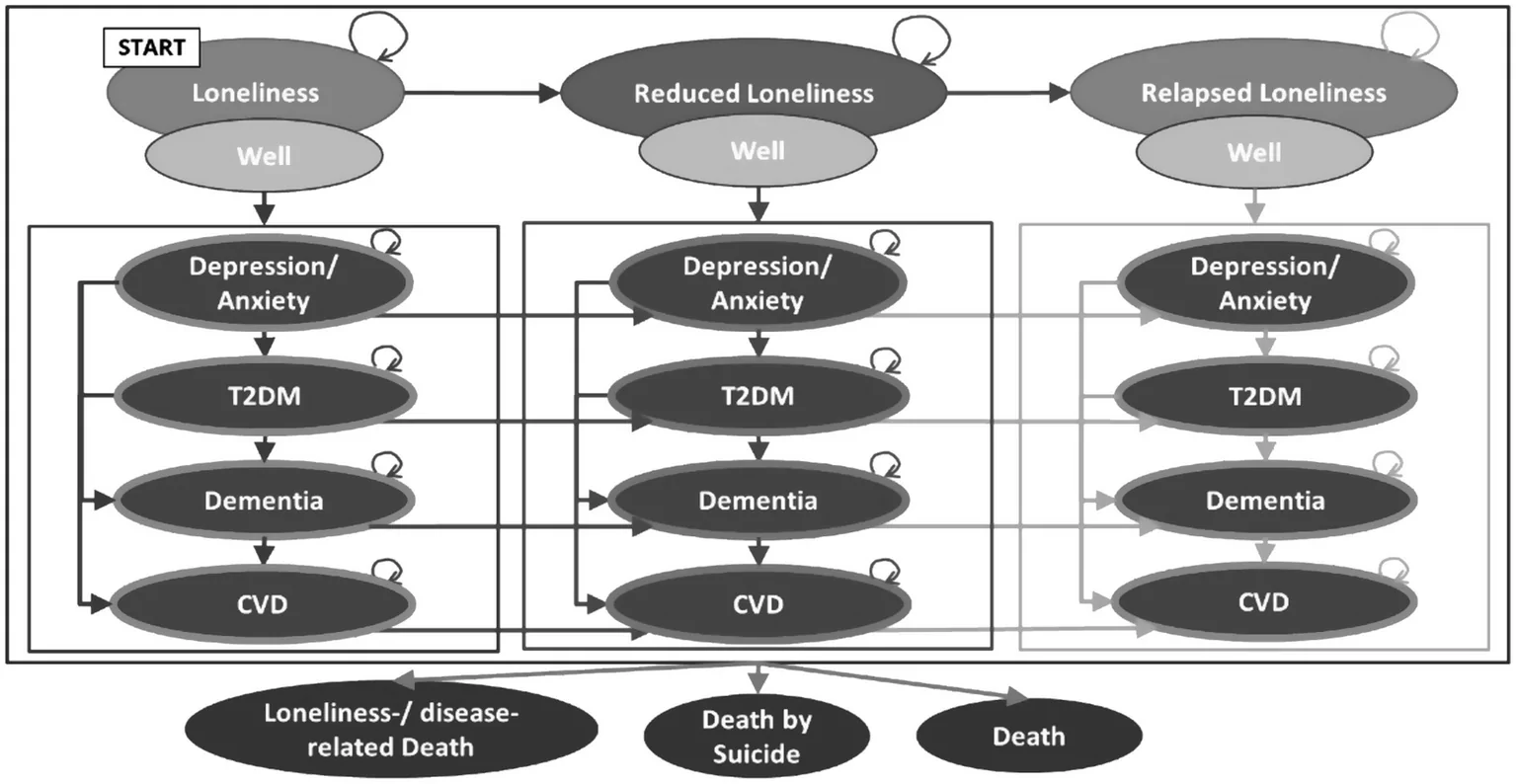
Structure of the Markov model. Ellipses represent health states and arrows indicate transition directions. All participants start in the “Loneliness & Well” state. After the first cycle, lonely participants can move to the “Reduced Loneliness & Well” state, stay in “Loneliness” or move to one of the loneliness-related disease states. Afterwards, participants can stay, relapse from “Reduced Loneliness” to “Relapsed Loneliness” once, with no possibility of moving to “Reduced Loneliness” again or move to the respective loneliness-related disease state. “Death” is possible from each health state. CVD: Cardiovascular diseases, T2DM: Diabetes mellitus type 2.

Health states were modeled as combinations of one of three loneliness conditions (i.e., “Loneliness”, “Reduced Loneliness”, “Relapsed Loneliness”), each paired with either a “well” state or a loneliness-related disease. Consistent with the trial, all participants started in the “Loneliness” state and were free of any disease. After the first cycle, lonely participants could move to “Reduced Loneliness”. Thereafter, participants could remain in “Reduced Loneliness” or relapse to “Relapsed Loneliness”. We explicitly used the term “Reduced Loneliness” instead of “No Loneliness” as participants might feel less lonely but still be classified as lonely according to the 11-item De Jong Gierveld Loneliness Scale (DJGLS) that was used to measure loneliness in the trial (de Jong-Gierveld and van Tilburg, 1999). Based on the DJGLS, a score of three to eleven indicates loneliness (de Jong-Gierveld and van Tilburg, 1999).

We identified health consequences related to loneliness and considered only diseases classified as such in the WHO’s advocacy brief (World Health Organization, 2021). In the model, anxiety, CVD, depression, dementia, T2DM, and death by suicide were included. Depression and anxiety were combined into a single health state. Other consequences of loneliness, such as behavioral actions (e.g., drug use, food intake, sleep hygiene, physical activity), intermediate steps/accidents (e.g., falls, fractures) or risk factors (e.g., high cholesterol, high blood pressure) were not considered. Participants could experience only one disease at a time and additional comorbidities were not considered. A hierarchy of the included loneliness-related diseases was constructed. The hierarchy of these diseases was established by ranking the health consequences of loneliness according to their impact on mortality in Spain. While progression to a more severe disease was possible, a return to a less severe disease was not. For example, it was possible to move from “Depression & Anxiety” to “CVD” but not in the opposite direction. The establishment of a hierarchy was necessary for two reasons. First, if people were to remain in the health state with their first disease, they might be prevented from progressing to more severe diseases. For example, suffering from “Depression & Anxiety” could prevent people from getting “CVD” in the model. Second, without a hierarchy and the possibility to move between all disease states at all time points, unrealistic scenarios could arise, such as having “CVD” for one year, “T2DM” the next, “CVD” again, then “Dementia”, and then “CVD” again. We assumed that a combination of diseases was not possible. The hierarchical classification of the diseases considered in the model ranges from “Depression & Anxiety” to “T2DM” to “Dementia” to “CVD”. Three absorbing states, “Death” from other causes, “Loneliness-/ disease-related Death” and “Death by Suicide”, were modeled to evaluate avoided loneliness-disease-related deaths and deaths by suicide.

The simulated cohort reflected the characteristics of the FiN trial in Barcelona, Spain, experiencing loneliness starting with the participants’ average age of 61 years and a gender distribution of 80% female and 20% male participants. Participants who were not lonely according to the DJGLS at baseline were excluded. The cohort entered the model in “Lonely & Well” and received either the NBSP intervention or AC in the first Markov cycle. A cycle length of three months was chosen to represent the duration of the NBSP intervention. At the end of each Markov cycle, the cohort could remain in the same health state or move to another health state with a given transition probability (transitions are shown as arrows in Figure 1). Cohort participants could remain in “Loneliness”, move to “Reduced Loneliness”, develop a disease, die from suicide or from other causes. If lonely and having a disease associated with loneliness, participants could remain in “Loneliness”, move to “Reduced Loneliness” within the respective disease, die from suicide or from other causes. Individuals in “Reduced Loneliness” can relapse to “Relapsed Loneliness” once, with no possibility of moving to “Reduced Loneliness” again as we assumed that this would require a second intervention.

We assumed that participants in “Reduced Loneliness” have the same risk of developing a specific disease or dying as participants not being lonely as no equivalent for “Reduced Loneliness” was found in the literature. Furthermore, we assumed that healthy individuals in the “Relapsed Loneliness” state have the same increased risk of developing a loneliness-related disease as those in the “Loneliness” state.

As loneliness is associated with long-term health consequences, the cohort is followed over the remaining lifetime horizon from a participant’s perspective to estimate the cohort’s expected health outcomes, measured in LYs, QALYs, and LFYs.

### Model inputs

Table 1 summarizes relevant information on the input parameters, corresponding sources, values, and distributions. All input parameters were converted to three month cycles for consistency within the model. Missing data within the Spanish RECETAS trial were addressed using multiple imputation by chained equations (MICE) (van Buuren and Groothuis-Oudshoorn, 2011). To address the potential bias from drop-out, inverse probability of censoring weights (IPCW) were estimated and applied to the analyses of the intervention effects, relapse probabilities and health state utility values (HSUV) (Cole and Hernan, 2008; Hernán and Robins, 2017).

**Table 1:** Input parameters and uncertainty parameters used in the model.

Type	Deterministic value	Uncertainty distribution	Deterministic SA (lower-upper)	Scenario analysis	Distribution parameters	Source
Discount rate	0.03	Fixed	-	SA 9	-	EUnetHTA (2015) and López-Bastida et al. (2010)
Average age (years)	61	Fixed	-	-	-	Trial Barcelona
Sex male	0.211	Fixed	-	-	-	
Sex female	0.789	Fixed	-	SA 4	-	
Time horizon	Lifelong	Fixed	-	SA 5	-	Assumption
Transition probability: AC from “loneliness” to “reduced loneliness”						
0 M-3 M	0.187	Beta	0.089–0.285	SA 1, SA 2, SA 3, SA 10	μ = 0.187, SE = 0.035	Trial
3 M-6 M	0.200	Beta	0.068–0.332	SA 1, SA 2, SA 3, SA 10	μ = 0.200, SE = 0.048	Trial
6 M-12 M	0.067	Beta	0–0.137	SA 1, SA 2, SA 3, SA 10	μ = 0.067, SE = 0.025	Trial
12 M+	0	Fixed	-	SA 6	-	Assumption
Relative risk: NBSP-FiN from “loneliness” to “reduced loneliness”						
Intervention effect 0 M-3 M	1.349	Lognormal	0.836–2.175	SA 1, SA 2, SA 3, SA 10	μ = 0.299, SE = 0.244	Trial
Intervention effect 3 M-6 M	1.042	Lognormal	0.526–2.066	SA 1, SA 2, SA 3, SA 10	μ = 0.041, SE = 0.349	Trial
Intervention effect 6 M-12 M	2.230	Lognormal	0.611–8.141	SA 1, SA 2, SA 3, SA 10	μ = 0.802, SE = 0.661	Trial
Intervention effect 12 M+	1	Fixed	-	SA 6	-	Assumption
Transition probability: AC from “reduced loneliness” to “relapsed loneliness”						
3 M-6 M	0.087	Beta	0–0.173	SA 1, SA 2, SA 3, SA 8, SA 10	μ = 0.087, SE = 0.031	Trial
6 M-12 M	0.144	Beta	0–0.344	SA 1, SA 2, SA 3, SA 8, SA 10	μ = 0.144, SE = 0.072	Trial
12 M+	0	Fixed	-	SA 6, SA 7	-	Assumption
Relative risk: NBSP-FiN from “reduced loneliness” to “relapsed loneliness”						
Relapse rate 3 M-6 M	1.364	Lognormal	0.486–3.827	SA 1, SA 2, SA 3, SA 8, SA 10	μ = 0.311, SE = 0.526	Trial
Relapse rate 6 M-12 M	0.627	Lognormal	0.131–2.992	SA 1, SA 2, SA 3, SA 8, SA 10	μ = −0.467, SE = 0.797	Trial
Relapse rate 12 M+	1	Fixed	-	SA 6, SA 7	-	Assumption
Transition probabilities to disease states for “well-reduced loneliness”						
“CVD”	Age- and gender specific	Fixed	-	-	-	Global Burden of Disease Collaborative Network (2022)
“Dementia”	Age- and gender specific	Fixed	-	-	-	
“Depression & Anxiety”	Age- and gender specific	Fixed	-	-	-	
“Diabetes”	Age- and gender specific	Fixed	-	-	-	
Risk to disease states for “loneliness”						
RR for “Dementia” if lonely	1.42	Lognormal	1.26–1.60	-	μ = 0.351, SE = 0.060	Wang et al. (2023)
RR for “T2DM” if lonely	1.41	Lognormal	1.04–1.90	-	μ = 0.344, SE = 0.154	Hackett et al. (2020)
RR for “CVD” if lonely	1.29	Lognormal	1.04–1.59	-	μ = 0.254, SE = 0.108	Valtorta et al. (2016)
OR for “Depression & Anxiety” if lonely	2.33	Lognormal	1.62–3.34	-	μ = 0.846, SE = 0.185	Mann et al. (2022)
Transition probabilities to death/suicide						
Background mortality	Age- and gender specific	Fixed	-	-	-	Global Burden of Disease Collaborative Network (2022)
Disease-specific mortality rate	Age- and gender specific	Fixed	-	-	-	
CFR for “CVD”	Age- and gender specific	Fixed	-	-	-	
CFR for “T2DM”	Age- and gender specific	Fixed	-	-	-	
CFR for “Dementia”	Age- and gender specific	Fixed	-	-	-	
RR for mortality with “Depression & Anxiety”	1.60	Lognormal	1.47–1.74	-	μ = 0.47, SE = 0.043	Walker et al. (2015)
Suicide	Age- and gender specific	Fixed	-	-	-	National Statistics Institute (2023)
OR for mortality if in “Loneliness”	1.26	Lognormal	1.04–1.53	-	μ = 0.231, SE = 0.098	Holt-Lunstad et al. (2015)
OR for suicide if in “Loneliness”	3.48	Lognormal	2.98–4.07	-	μ = 1.247, SE = 0.080	Alothman et al. (2024)
OR for suicide if in “CVD”	1.44	Lognormal	1.08–1.93	-	μ = 0.365, SE = 0.148	Chen et al. (2023)
OR for suicide if in “T2DM”	1.65	Lognormal	0.95–2.85	-	μ = 0.501, SE = 0.280	Wang et al. (2017)
OR for suicide if in “Dementia”	1.30	Lognormal	0.81–2.10	-	μ = 0.262, SE = 0.796	Desai et al. (2024)
HR for suicide if in “Depression & Anxiety”	1.58	Lognormal	1.25–1.99	-	μ = 0.457, SE = 0.119	Li et al. (2022)
Health-related quality of life utility values						
Utility for “Loneliness”	0.692	Beta	0.675–0.708	-	μ = 0.692, SE = 0.008	Trial Barcelona, EQ-5D-5L (Spanish value set)
Utility for “Reduced Loneliness”	0.764	Beta	0.739–0.790	-	μ = 0.764, SE = 0.012	Trial Barcelona, EQ-5D-5L (Spanish value set)
Utility for “Death”	0	Fixed	-		-	Assumption
Utility reduction factor for “Dementia”	0.638	Beta	0.49–0.54	-	Dementia: μ = 0.519, SE = 0.013	CEAFA (2022), Garcia-Gordillo et al. (2016), and Olazarán et al. (2017)
		Beta	0.8091–0.8189		Spain Population Norm: μ = 0.814, SD = 0.25, SE = 0.0049	Garre-Olmo et al. (2017)
Utility reduction factor for “CVD”	0.811	Beta	0.85–0.87	-	Angina: μ = 0.86, SE = 0.006, w = 40%	Hainsworth et al. (2024)
		Beta	0.725–0.795	-	PAD: μ = 0.76, SE = 0.018, w = 39%	
		Beta	0.79–0.83	-	Stroke: μ = 0.81, SE = 0.010, w = 21%	
Utility reduction factor for “T2DM”	0.907	Beta	0.7826–0.8224	-	Diabetes: μ = 0.803, SE = 0.0101	Collado Mateo et al. (2015), Konerding et al. (2017), and Garcia-Gordillo et al. (2016)
			0.878–0.892	-	Population Norm: μ = 0.885, SE = 0.0036	
Utility reduction factor for “Depression & Anxiety”	0.814	Beta	−0.184 – −0.150	-	Disutility: μ = 0.167, CI, 0.184–0.150	Sabes-Figuera et al. (2012)
		Beta	-	-	Population norm: μ = 0.90, SE = 0.001	

For lognormal distributions, the reported mean and standard error refer to the parameters on the natural log scale; base-case model inputs were based on the distributional mean of the fitted distributions (Briggs et al., 2006a). AC: Active control, CFR: Case Fatality Rate, CI: 95% Confidence Interval, CVD: Cardiovascular Diseases, FiN: Friends in Nature, HR: Hazard Ratio, M: Month, NBSP: Nature-based Social Prescribing, OR: Odds Ratio, RR: Relative Risk; SA: Sensitivity Analysis, SE: Standard Error, T2DM: Diabetes mellitus type 2, w: Weight, μ: Mean.

#### Intervention effect and relapse probability

Time-dependent intervention effects and relapse probabilities were calculated based on trial data from Barcelona. In the trial, loneliness was measured with the DJGLS, ranging from 0 to 11 (de Jong-Gierveld and van Tilburg, 1999). The intervention effect and relapse probabilities were estimated using modified Poisson regression over the weighted pseudo-population for each of the five imputations and pooled using Rubin’s rule (Rubin, 1987). We assumed that all participants missing due to drop-out would have failed to improve or relapse between the respective time points. Based on expert consensus, the intervention was considered successful, meaning moving to “Reduced Loneliness”, if a participant’s score decreased by two or more points on the DJGLS. All participants whose DJGLS score increased again by two or more points, were considered in “Relapsed Loneliness”. Although cutoff points for the DJGLS exist to differentiate between not lonely, moderately lonely, and strongly lonely (de Jong-Gierveld and van Tilburg, 1999), we determined a specific point change as a meaningful improvement to account for individual differences in the baseline loneliness score and acknowledge an improvement in those who would still be classified as lonely according to the scale. After the maintenance phase of one year, no intervention effect and relapse probability were applied.

#### Development of loneliness-related diseases

Spanish age- and gender specific incidence rates for anxiety, CVD, depression, dementia, and T2DM were retrieved from the Institute for Health Metrics and Evaluation (IHME) (Global Burden of Disease Collaborative Network, 2022) and applied to individuals in “Reduced Loneliness”. To determine the impact of loneliness on the incidences of the included diseases, effect size parameters comparing non-lonely with lonely individuals were derived from published literature and implemented as multipliers in the model (Hackett et al., 2020; Valtorta et al., 2016; Wang et al., 2023; Wang et al., 2018). We included a relative risk (RR) of 1.29 (95% Confidence Interval (CI): 1.04–1.59) for CVD (Valtorta et al., 2016), a RR of 1.42 (CI: 1.26–1.60) for developing dementia (Wang et al., 2023), an odds ratio (OR) of 2.33 (CI: 1.62–3.34) for depression & anxiety (Mann et al., 2022), and a hazard ratio (HR) of 1.41 (CI: 1.04–1.90) for T2DM in lonely versus non-lonely individuals (Hackett et al., 2020).

#### Mortality

Spanish all-cause mortality and cause-specific mortality rates by age- and gender for CVD, dementia, and T2DM were derived from the IHME database (Global Burden of Disease Collaborative Network, 2022). Age and gender-specific suicide rates were retrieved from the Spanish National Statistics Institute (INE) (National Statistics Institute, 2026). We calculated age- and gender-specific case fatality rates (CFR) for CVD, dementia, and T2DM using data from the IHME database (Global Burden of Disease Collaborative Network, 2022). For mortality associated with depression and anxiety, a RR of 1.60 (CI, 1.47–1.74) was applied to all-cause mortality (Walker et al., 2015). We also considered the impact of loneliness on mortality and on suicide risk as well as the effect of diseases on suicide risk. Excess mortality for lonely individuals was calculated using the OR of 1.26 (CI, 1.04–1.53) for increased mortality from loneliness (Holt-Lunstad et al., 2015). The OR of 3.48 (CI, 2.98–4.07) for loneliness-related suicide (Alothman et al., 2024), the HR of 1.58 (CI, 1.25–1.99) for suicide due to anxiety and depression (Li et al., 2022), the OR of 1.44 (CI, 1.08–1.93) due to CVD (Chen et al., 2023), the OR of 1.65 (CI, 0.95–2.85) due to T2DM (Wang et al., 2017), and the OR of 1.30 (CI, 0.81–2.10) due to dementia (Desai et al., 2024) were integrated into our model. To avoid double-counting deaths in disease states (Weinstein et al., 2003), we corrected the all-cause mortality for the disease-specific mortality, death by suicide and the excess mortality associated with loneliness.

#### Health-related quality of life

Health-related quality-of-life (HRQoL) was measured using HSUVs ranging from 0 (dead) to 1 (perfect health) (Roudijk et al., 2022). For the loneliness states, HSUVs were obtained from the Barcelona trial where the generic quality-of-life instrument, EQ-5D-5L questionnaire (EuroQol Research Foundation, 2019), was applied using the Spanish tariff (Ramos-Goñi et al., 2018). HSUVs were used to calculate QALYs (Devlin et al., 2020). A mean utility value of 0.69 over all timepoints when trial participants were classified as lonely according to the DJGLS was used for “Loneliness” and “Relapsed Loneliness”. The utility value for “Reduced Loneliness” was derived from participants whose DJGLS score decreased by two or more points. The average of all values in “Reduced Loneliness” of all individuals was used, resulting in a mean utility of 0.76. Utility reduction factors for the modeled loneliness-related diseases in a Spanish population were derived and applied as multipliers to the utility value for “Loneliness” or “Reduced Loneliness”. The following utility decrements were applied: 0.64 for dementia (Garcia-Gordillo et al., 2016; Garre-Olmo et al., 2017; Olazarán et al., 2017), 0.81 for CVD (Hainsworth et al., 2024), 0.91 for T2DM (Garcia-Gordillo et al., 2016; Collado Mateo et al., 2015; Konerding et al., 2017), and 0.81 for depression and anxiety (Sabes-Figuera et al., 2012). Derivation and calculation of all utility values are presented elsewhere (Rochau et al., 2026).

#### Base-case analysis

In the base-case analysis, we estimated LYs, QALYs and LFYs for the study population described above receiving NBSP or AC. LYs quantify the additional life expectancy, whereas QALYs combine life expectancy and quality of life (Drummond et al., 2015). LFYs reflect time with reduced feelings of loneliness. Time spent in each “Loneliness” state was assigned a value of 0, whereas time spent in each “Reduced Loneliness” state was assigned a value of 1. A half-cycle correction was applied to initial and final health outcomes. Economic outcomes were not included and discounting was not applied in the base-case analysis. Additionally, we compared loneliness- disease-related deaths in the NBSP strategy against AC. The model was programmed and validated in the software TreeAge Pro Healthcare 2023 (TreeAge, n.d.).

### Sensitivity analyses

We performed deterministic and probabilistic sensitivity analyses (PSA) to assess the robustness of the models’ outcomes. For deterministic sensitivity analyses, input parameter ranges were based on the 95% CIs. Tornado diagrams were used to show the parameters with the strongest impact on the outcome. In the PSA, we performed a Monte Carlo simulation with 10,000 iterations for the base-case scenario to assess parameter uncertainty (Hatswell et al., 2018). Parameters were assigned their most likely probability distributions: utility parameters and transition probabilities were drawn randomly from a beta distribution, while RRs, HRs and ORs were sampled from a lognormal distribution (Briggs et al., 2006b). Table 1 presents the lower and upper limits of the input parameters and their distributions. Equal-tailed 95% credible intervals (CrI) were used to summarize posterior uncertainty, reflecting the 95% range of the posterior distribution of model-generated results (Hespanhol et al., 2019).

### Scenario analyses

We performed eleven scenario analyses (SA) to explore the impact of alternative assumptions. Instead of defining effectiveness as a reduction of two or more points on the DJGLS, we defined effectiveness for NBSP and AC as a score below three (not lonely), with relapse defined as an increase to three or more (SA 1). We adjusted for baseline imbalances between the groups (SA 2). To assess intervention effects, relapse probabilities and the utility multiplier for “Reduced Loneliness” directly from the observed population data, results were evaluated without IPCW and adjustments for baseline imbalances (SA 3). We examined the effect in a female-only cohort (SA 4). We changed the time horizon of the analysis from lifetime to a duration of 1, 5, or 10 years (SA 5) (EUnetHTA, 2015). While the base-case assumed an intervention effect and a relapse probability over one year, we extended both for five years (SA 6). Based on estimates from RECETAS experts, we extended the period in which relapse is possible by one additional year while keeping the intervention effect the same as in the base-case analysis (SA 7). We modeled a conservative scenario in which, due to the wide margin of uncertainty, we did not assume a relative intervention effect or a relapse probability for NBSP-FiN at 6 months (SA 8). A scenario analysis with a discount rate of 3% per year was performed to consider a lower valuation of future events and conditions (SA 9). We conducted a complete case analysis including only participants for whom all loneliness scores at baseline, 3, 6, and 12 months were available (*n* = 139) (SA 10). In contrast to the base-case analysis, which assumed that all participants who dropped out failed to improve or to relapse, missing outcomes due to drop-out were retained as missing and the intervention effect and relapse probability were calculated only among participants who remained under observation, reweighted according to their probability of being observed (SA 11).

### Model validation

Following international modeling guidelines from the ISPOR-SMDM Modeling Good Research Practices Task Force (Eddy et al., 2012), we assessed the concept of the decision-analytic model and input parameters (i) for face validity with experts in decision modeling and RECETAS trial collaborators with expertise in loneliness and its consequences for health and wellbeing, (ii) internal validity against the TECHnical VERification (TECH-VER) checklist (Buyukkaramikli et al., 2019), and (iii) external validity against empirical evidence on life expectancy. We reported our validation activities using the Validation-Assessment Tool of Health-Economic Models for Decision Makers and Model (AdViSHE) for health-economic models (Vemer et al., 2016).

## Results

### Model validation

Face validity was assessed through expert review of the model structure, assumptions, input parameters, and transition pathways. Internal validity was independently assessed using the TECH-VER checklist, including checks of input parameters, formulas, links, and calculations for consistency and correctness. External validation was assessed by comparing model outcomes with independent published results. A slightly higher life expectancy than indicated in national statistics may be explained by the fact that all participants entered the model as lonely and free of disease. In practice, a proportion of 61-year-olds are likely to already have underlying health conditions. More detailed results of the model validation can be found in Appendix 1. The TECH-VER, AdViSHE, and CHEERS checklists are provided upon request (Husereau et al., 2022; Buyukkaramikli et al., 2019; Vemer et al., 2016).

### Base-case analysis

The results of the NBSP intervention are presented in Table 2. Over a lifetime horizon, NBSP was shown to be more beneficial than AC in terms of LYs, QALYs and LFYs. With a starting age of 61 years, NBSP participants achieved on average 23.98 LYs, 15.15 QALYs, and 10.73 LFYs, whereas AC participants achieved 23.68 LYs, 14.76 QALYs, and 8.11 LFYs. NBSP increased life expectancy by 0.30 years, yielded additional 0.39 QALYs, and 2.62 LFYs. Our model indicated that NBSP could avert about 1.5% of loneliness-disease-related deaths compared to AC.

**Table 2:** Results of the base-case and scenario analyses.

		LYs		QALYs		LFYs	
		Total	Incremental	Total	Incremental	Total	Incremental
Base-case analysis	NBSP	23.98	0.30	15.15	0.39	10.73	2.62
	AC	23.68		14.76		8.11	
SA 1—Not Lonely as per DJGLS threshold of three	NBSP	23.67	0.59	15.36	1.07	8.03	5.14
	AC	23.08		14.29		2.89	
SA 2—With adjustments for baseline imbalances	NBSP	23.98	0.30	15.15	0.39	10.71	2.60
	AC	23.68		14.76		8.11	
SA 3—Without IPCW, without adjustments for baseline imbalances	NBSP	23.99	0.26	15.13	0.34	10.74	2.22
	AC	23.73		14.79		8.52	
SA 4—Female cohort only	NBSP	25.04	0.27	15.42	0.38	11.35	2.72
	AC	24.77		15.04		8.63	
SA 5—Time horizon over 1 year	NBSP	1	0	0.69	0.01	0.31	0.06
	AC	1		0.68		0.25	
SA 5—Time horizon over 5 years	NBSP	4.94	0	3.33	0.04	2.02	0.48
	AC	4.94		3.29		1.54	
SA 5—Time horizon over 10 years	NBSP	9.69	0.01	6.34	0.10	4.09	0.98
	AC	9.68		6.24		3.11	
SA 6—Effects over 5 years	NBSP	23.78	0.35	14.92	0.46	9.45	3.18
	AC	23.43		14.46		6.27	
SA 7—Relapse for one additional year	NBSP	23.71	0.27	14.80	0.34	8.36	2.28
	AC	23.44		14.46		6.08	
SA 8—Conservative scenario	NBSP	23.79	0.11	14.91	0.15	9.05	0.94
	AC	23.68		14.76		8.11	
SA 9—Discounting	NBSP	16.50	0.15	10.57	0.23	7.21	1.76
	AC	16.35		10.34		5.45	
SA 10—Complete case analysis	NBSP	24.18	0.18	15.40	0.24	12.52	1.64
	AC	24.00		15.16		10.88	
SA 11—Effects among non-drop-outs	NBSP	24.12	0.16	15.34	0.22	11.95	1.45
	AC	23.96		15.12		10.50	

Increments are based on table values rounded to two decimal places. AC: Active control, DJGLS: De Jong Gierveld Loneliness Scale, IPCW: Inverse probability of censoring weights, LFYs: Loneliness-free years, LYs: Life Years, NBSP: Nature-based social prescribing, QALYs: Quality-adjusted life years; SA: Scenario analysis.

### Sensitivity analyses

Figure 2 illustrates the deterministic sensitivity analysis for the health outcomes (LYs, QALYs and LFYs). The tornado diagrams indicate that model outcomes were most sensitive to assumptions regarding the effectiveness of AC and NBSP in reducing loneliness after 6–12 months, relapse during this period, and the increased mortality risk associated with loneliness. Figure 3 presents the results and probability distributions of LYs, QALYs and LFYs for NBSP and AC across 10,000 Monte Carlo simulations. NBSP was the preferred strategy in 89% of all simulations for QALYs, LYs, or LFYs. With NBSP, participants gained 0.29 (CrI: −0.15–1.02) LYs, 0.37 (CrI: −0.19–1.15) QALYs, and 2.48 LFYs (CrI: −1.26–7.19).

**Figure 2:**
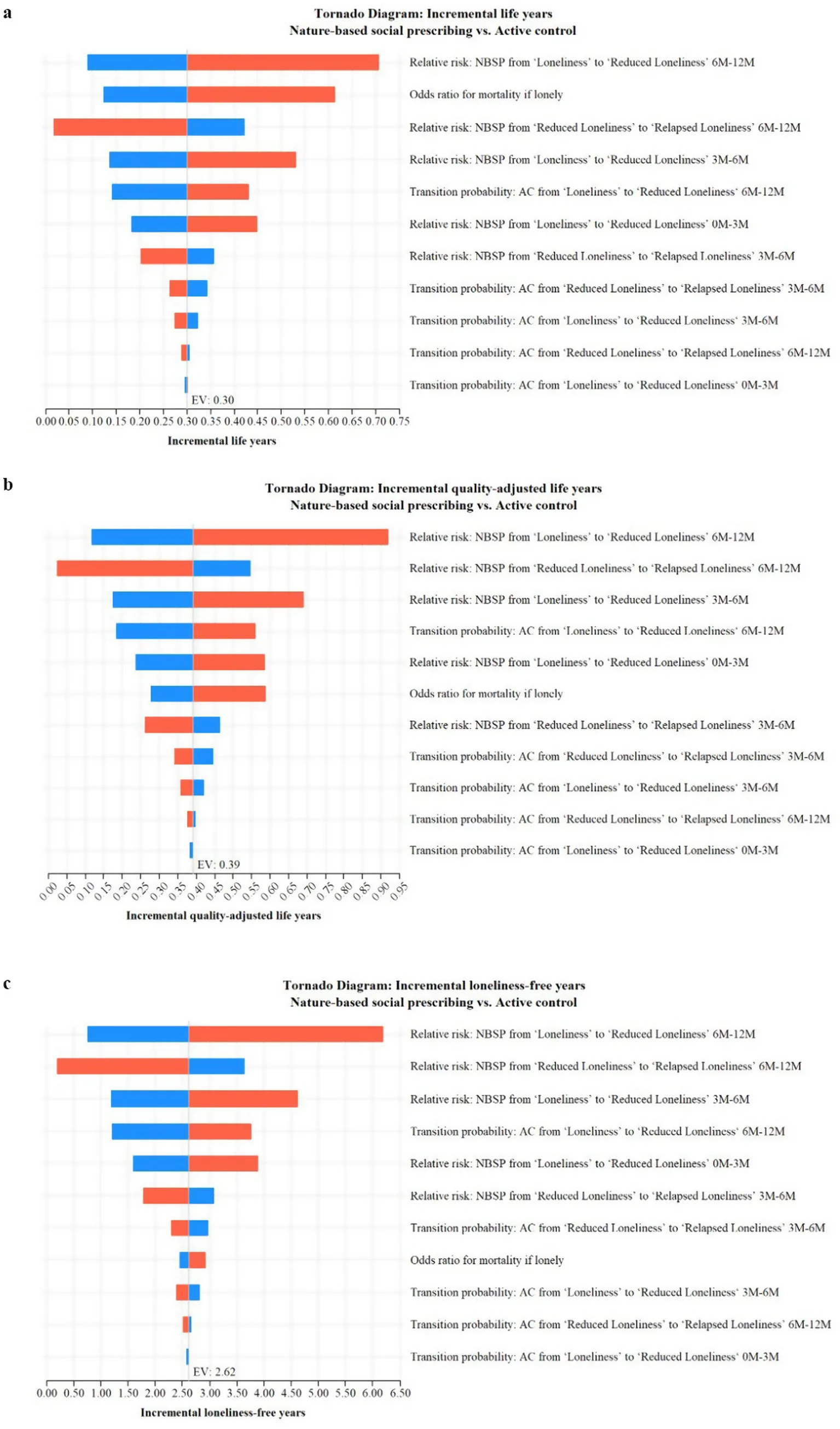
Tornado diagrams showing the input parameters with the highest impact on life years **(a)**, quality-adjusted life years **(b)**, and loneliness-free years **(c)**. Blue bars represent the change in the incremental health outcome when each input parameter varies from its lower bound to the base case value; Red bars represent the change from the base-case value to the upper bound, showing which parameter increases or decreases the incremental health outcome.

**Figure 3:**
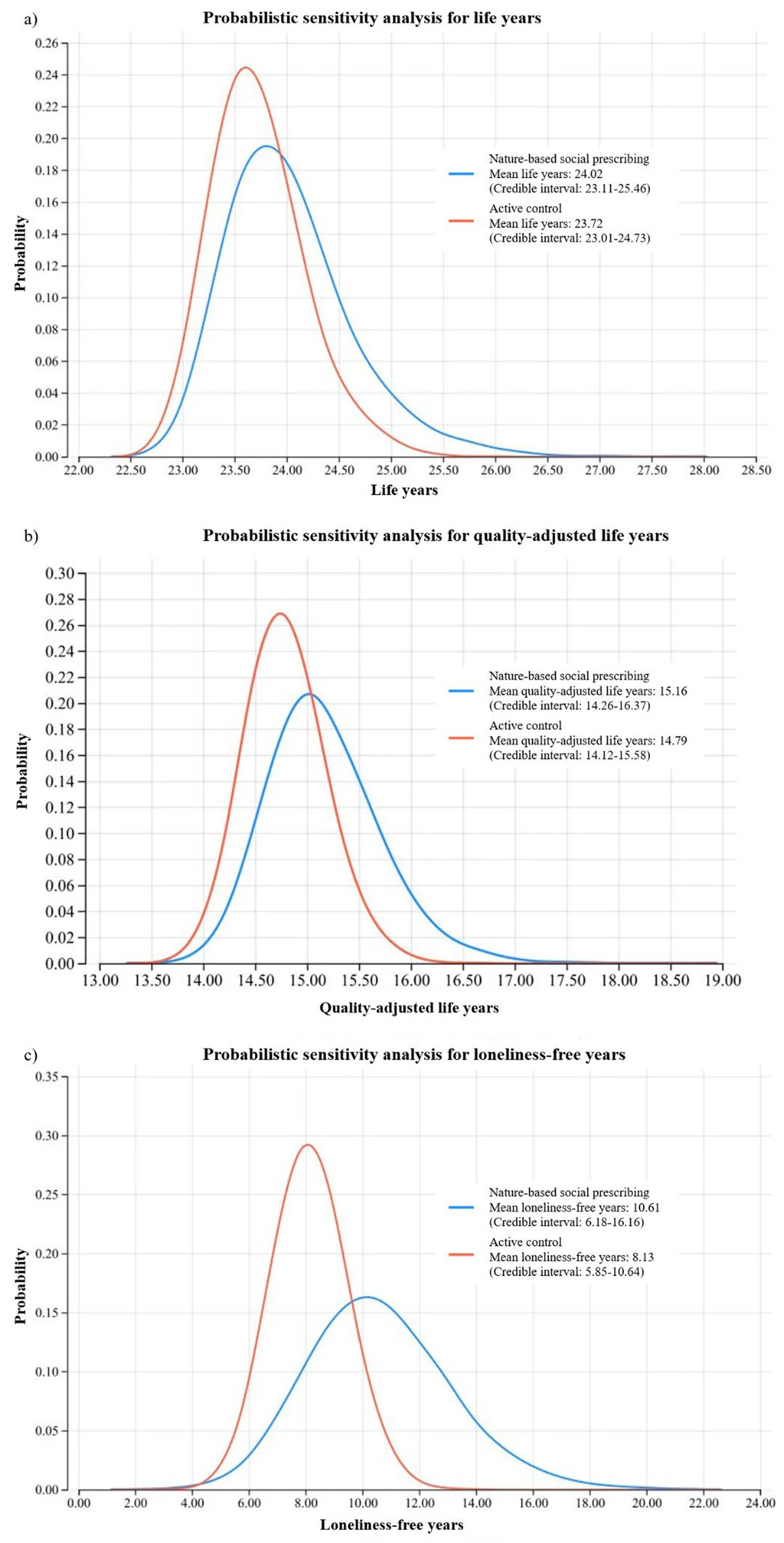
Probabilistic sensitivity analysis for life years **(a)**, quality-adjusted life years **(b)** and loneliness-free years **(c)**. Results and probability distributions of life years, quality-adjusted life years, and loneliness-free years for nature-based social prescribing and active control across 10,000 Monte Carlo simulations.

### Scenario analyses

The results remained robust across the scenario analyses (see Table 2). Using the DJGLS threshold of three for defining intervention effectiveness and relapse (SA 1), NBSP became more effective than AC, especially after 6 and 12 months, resulting in higher incremental outcomes in favor of NBSP. Adjusting for baseline imbalances had a minor effect on incremental outcomes (SA 2). Estimating effects without IPCW and without adjustments yielded slightly less incremental LYs, QALYs, and LFYs for NBSP (SA 3). In a female-only cohort (SA 4), NBSP and AC were associated with slightly more QALYs, LYs, and LFYs, consistent with the life expectancy in Spain. Following the cohort over shorter time horizons (SA 5) revealed almost no incremental LYs between NBSP and AC after 1, 5 and 10 years. In contrast, incremental QALYs in favor of NBSP were already observed after 5 years (0.04) and became more obvious after 10 years (0.10). A small difference in LFYs was evident already after 1 year and increased at 5 years and 10 years. If the effect of the intervention and the possibility to relapse lasted more than five years (SA 6), health gains decreased for both strategies. Extending only the duration of the relapse probability by one additional year (SA 7), incremental health gains declined slightly, still in favor of NBSP. Assuming a conservative scenario, considering the high uncertainty around 6–12 months effectiveness and relapse data (SA 8), health outcomes would be less for both strategies, with less incremental outcomes in favor of NBSP than in the base-case. Applying discounting demonstrated a lower valuation of future health outcomes (SA 9). Using only complete cases, total health outcomes increased for both groups (SA 10). Deriving intervention effects and relapse probabilities without the assumption that all participants who dropped out failed to improve or to relapse, absolute health outcomes would be higher for NBSP and AC. Incremental effects decreased but were still in favor of NBSP (SA 11).

## Discussion

We evaluated the long-term impacts of NBSP as a loneliness-reduction strategy among older adults in Spain. Based on our results, NBSP is the more effective strategy, as 0.30 additional LYs, 0.39 additional QALYs and 2.62 additional LFYs were gained compared to AC.

Our findings align with the results of prior modeling studies evaluating strategies to reduce loneliness (McDaid and Park, 2021; Engel et al., 2021) that assume group-based interventions to be effective. However, to the best of our knowledge, no long-term decision-analytic model evaluating the health outcomes of a nature-based social prescribing intervention to alleviate loneliness has been published.

A key limitation of previous studies evaluating loneliness-alleviating strategies is that they did not derive effectiveness from RCTs (McDaid and Park, 2021; Engel et al., 2021). In contrast, we derived evidence from a RCT conducted in Barcelona. Moreover, both previous studies evaluating loneliness-reduction strategies only applied a time period of five years (McDaid and Park, 2021; Engel et al., 2021). Additionally, Engel et al. did only consider depression and suicide attempt as health consequence of loneliness (Engel et al., 2021). However, loneliness is associated with more long-term health consequences. We also applied a Markov state-transition cohort model, considering a broader set of loneliness-related diseases, and simulated a life-long time horizon, with the possibility for the cohort to relapse once to “Relapsed Loneliness”. Although our results show that life expectancy gains are small for the NBSP participants, their remaining quality of life could be improved, and importantly, participants could gain more than 2.6 additional years without loneliness. The long-term impacts of reducing loneliness with NBSP were confirmed by scenario analyses over shorter time horizons. As there is almost no difference between both groups after one year, the effects become more apparent after ten years. We also showed the importance of maintaining the intervention effect from baseline to 3 months, as reductions in intervention effects may be offset by a relapse to loneliness. As expected, and consistent with the Spanish gender-specific life expectancy (National Statistics Institute, 2023), restricting the analysis to females resulted in more LYs and, correspondingly, more QALYs and LFYs for both strategies.

Incremental health outcomes remained stable in deterministic sensitivity analysis, favoring NBSP over AC. The relative intervention effect and relapse probability have a notable impact on health outcomes. As these values were derived directly from the trial, we consider them appropriate estimates for our model. However, effectiveness and relapse parameters had a high uncertainty. Additionally, the increased mortality associated with loneliness had a strong impact on health outcomes; this parameter was derived from a meta-analysis explicitly excluding suicide and adjusting for several covariates including depression (Holt-Lunstad et al., 2015). However, the estimated mortality effect may still partially overlap with mortality associated with loneliness-related diseases already included in our DAM, potentially resulting in residual double-counting and overestimation of loneliness-related mortality. Although correction procedures were applied, this potential overlap cannot be fully excluded and should therefore be considered when interpreting the estimated health outcomes. Results of the PSA demonstrated that NBSP is superior to AC even under parameter uncertainty, as NBSP was identified to be the preferred strategy in 89% of all simulations for all health outcome QALYs, LYs, or LFYs. However, the incremental credible intervals for LYs, QALYs, and LFYs included zero, indicating uncertainty around the magnitude and direction of the estimated benefits.

### Limitations

Our modeling study has several limitations. The model was informed by RCT data that contained missing values and drop-outs. For the calculation of the intervention effect and relapse probability, we assumed that participants who dropped out would have failed to improve or relapse between the respective time points. Due to differential drop-out, this overestimates the intervention effect and underestimates the relapse probability in the NBSP group. However, deriving effects only from non-drop outs, reweighted according to their probability of being observed, between the respective time points showed that NBSP remains favorable strategy in SA 11.

Complete outcome data were available for only 139 participants (FiN: 83, AC: 56) across the three, six and 12-month follow-ups, which increases uncertainty and the potential impact of missing data assumptions. However, for transparency, we conducted a scenario analysis for complete cases showing that the incremental health outcomes were still in favor of NBSP. Moreover, by combining MICE with IPCW, we aimed to overcome incomplete data to derive unbiased estimates of the intervention effect, relapse probability and HSUV. Baseline imbalances were addressed in a sensitivity analysis by controlling for potential measured confounders. Although multiple imputation, inverse probability of censoring weighting, and complete-case analyses showed consistent findings, these approaches cannot fully exclude bias related to unobserved factors associated with missing outcomes. Most likely, results are biased due to missing data and differential drop-outs (Coll-Planas et al., 2026).

We assumed a threshold approach for “Loneliness” or “Reduced Loneliness” without intermediate states, such as moderate or severe loneliness. In the “Reduced Loneliness” state, disease progression was parameterized using risk estimates for individuals who were not lonely. Although participants were in a state of “Reduced Loneliness”, we used the terminology of loneliness-free years, which should be considered when interpreting the resulting LFYs. However, this is a conservative assumption biasing towards AC as seen in SA 1.

Furthermore, relapse was only possible in the first year. SA 7 showed a slight decrease in incremental effects with prolonged relapse probability. Therefore, our assumption might favor the intervention. Additionally, the effects of a second intervention and subsequently a possible repeated relapse on resulting LYs, QALYs, and LFYs were not explored to reflect the RECETAS trial as accurately as possible. In reality, however, individuals could take part in a second intervention to reduce their feelings of loneliness.

We constructed a hierarchical structure of diseases based on the impact on mortality in Spain, restricting progression to a single direction and only allowing progression to more severe diseases. For example, dementia was not explicitly modeled for individuals with CVD, even though some individuals with CVD may develop dementia. Therefore, the model did not allow any of the loneliness-related diseases to happen at the same time and did also not consider further comorbidities. This might lead to an underestimation of the diseases burden. On the other hand, we did not allow any cure of the diseases, which might overestimate the disease burden. Implicitly, comorbidities may be included in some disease-related utility values as the respective study participants may have suffered from comorbidities. Additionally, transition probabilities derived from the FiN trial in Barcelona included a heterogenous population. In our model, all participants started in “Loneliness” and were free of any disease. However, participants in the trial might not have been disease-free and possibly experienced varying degrees of loneliness. Thus, the modeled life expectancy differed from national estimates, which reflects the general population and its underlying distribution of health conditions and risk factors. Further, we did not consider that individuals in “Reduced Loneliness” have a higher mortality due to their history of loneliness. We also assumed that international data for effect sizes comparing not lonely with lonely individuals were appropriate for the Spanish context. For example, the impact of loneliness on diseases was retrieved from meta-analyses conducted outside of Spain. Additionally, we assumed that effect measures derived from meta-analyses were constant and applicable beyond the mean follow-up of the studies. Another limitation is that our results may not be generalized beyond the Spanish context. Within the RECETAS project, we evaluated FiN, a NBSP intervention adapted to local conditions in Barcelona, Spain, and for a specific target population. Our findings may not be directly applicable to other populations living in other countries, having a different age, or socioeconomic status. When implementing this intervention in another region and to another target population, adaptations to the NBSP intervention should be considered, for example for the Finish context as presented by Pitkala et al. (2026).

While our results indicated that NBSP increases health benefits, social prescribing may also entail potential harms or unintended negative consequences, such as stigmatization due to loneliness, exclusion of specific groups, or added stress for healthcare professionals managing challenging group dynamics and participant behavior (Papon et al., 2026; Cooper et al., 2026). Although QALYs might already include harms, further research should specifically include such mentioned harms into quantitative decision analyses to assess and explicitly report quantitative benefit-harm balances of NBSP. Additionally, future studies could investigate the impact of uncertainty as highlighted by the PSA, and extend this work through value-of-information analyses to identify priorities for and extent of future research and reduce decision uncertainty (Ades et al., 2004; Sculpher and Claxton, 2005). Future research should also integrate costs of the intervention and loneliness-related health consequences to support decision makers whether NBSP is cost effective in the long term. Importantly, further research should consider the scenario analyses depending on the specific research question and underlying assumptions.

## Conclusion

We developed and validated a decision-analytic model to evaluate the long-term effectiveness of FiN, an NBSP intervention, compared to AC in alleviating loneliness and its impact on life expectancy, quality-adjusted life expectancy and loneliness-free time. We extended the one-year follow-up of the RECETAS-BCN RCT by linking observed short-term loneliness states with projected long-term health consequences of loneliness and mortality. Our findings suggest potential long-term benefits of NBSP to alleviate loneliness among adults in Spain. Although the long-term outcomes are model-based projections dependent on model assumptions and external epidemiological evidence and incredible intervals indicated uncertainty around the magnitude and direction of the estimated benefits, our findings provide healthcare policy decision-makers with relevant information on the potential value of NBSP for reducing loneliness when no long-term empirical data are available.

## Data Availability

The datasets presented in this article are not readily available because the RECETAS trial data analyzed in this study was obtained through a data transfer agreement between ISGlobal and UMIT TIROL. Requests to access the datasets should be directed to JL, jill.litt@isglobal.org.

## Glossary

AC: active control
AdViSHE: Validation-Assessment Tool of Health-Economic Models for Decision Makers and Model
CFR: case fatality rates
CHEERS: Consolidated Health Economic Evaluation Reporting Standards
CI: confidence interval
CrI: credible interval
CVD: cardiovascular diseases
DJGLS: De Jong Gierveld Scale
FiN: Friends in Nature
HR: hazard ratio
HRQoL: health-related quality of life
IHME: Institute for Health Metrics and Evaluation
INE: National Statistics Institute
IPCW: inverse probability of censoring weights
LFYs: loneliness-free years
LY: life years
MICE: Multiple Imputation by Chained Equations
NBSP: nature-based social prescribing
OR: odds ratio
PSA: probabilistic sensitivity analysis
QALYs: quality-adjusted life years
RCT: randomized controlled clinical trial
RECETAS: Re-imagining Environments for Connection and Engagement: Testing Actions for Social Prescribing in Natural Spaces
RR: relative risk
SA: scenario analysis
SP: Social Prescribing
T2DM: diabetes type 2
TECH-VER: TECHnical VERification
WHO: World Health Organization
