# Supplementary material for: Comparative effectiveness of nature-based social prescribing to alleviate loneliness in Spain: a decision analysis informed by the RECETAS trial
Source: Front Psychol. 2026 Oct 8;17:1891830. doi: 10.3389/fpsyg.2026.1891830 (PMC13687080; doi:10.3389/fpsyg.2026.1891830)
Supplement: Supplementary file 1 [file Data_Sheet_1.pdf]

Supplementary Online Appendix

Appendix 1 – Technical appendix

This appendix provides details on the statistical analysis of the RECETAS trial data as well as the derivation of decision-analytic model input parameters. Additionally, health states, transitions and transition probabilities are presented, followed by a paragraph on validation of the decision-analytic model.

Statistical Analysis of the RECETAS trial data

Missing data

Amongst others, health-related quality of life measured with the EQ-5D-5L [1], and loneliness with the De Jong Gierveld Scale (DJGLS) [2] ranging from 0 to 11, categorizing loneliness into three stages (0-2: not lonely, 3-8: moderately lonely, 9-11: strongly lonely), were assessed in the RECETAS trial in the nature-based social prescribing (NBSP) intervention, Friends-in-Nature (FiN), and active control (AC) group. Baseline EQ-5D items and DJGLS items were complete.

For follow-up time points, we observed that missingness followed more a unit non-response instead of item non-response [3, 4] as mostly the entire instrument was missing and seldom individual items. Regression of missing EQ-5D and DJGLS variables at three month on the randomized group, baseline EQ-5D/ DJGLS, sociodemographic and wellbeing variables showed that the group was a significant predictor for missingness. Missing values are unlikely to be missing completely at random (MCAR), instead it can be assumed that missing data is missing at random (MAR), meaning depending on values of the observed variables [5]. Multiple imputation (MI) can be applied to address unit and item nonresponse following MAR pattern [5, 6]. Missing EQ-5D-5L and DJGLS data during follow-up were addressed using multiple imputation by chained equations (MICE) [7]. With missingness of more than 50% of the questionnaire items, the score was imputed. With missingness less than 50% of the questionnaire items, each missing item was imputed. Although, the "total loneliness score is valid only if the sum of the missing […] equals 0 or 1" [8], we performed item level imputation if the amount of missing items was less than or equal to 6 for each timepoint. If we would have followed the original rules, the high number of missing items would mean omitting a lot of valuable information. Multiple imputation with one imputation model was performed for both groups separately [7]. For the imputation, predictors were sex, age, area, employment status, education, baseline EQ-5D score, baseline capability score, and baseline loneliness score. Proportional odds logistic regression was set as an imputation method for ordinal items. Scores were imputed with

predictive mean matching. The imputation was run five times to create five imputed datasets. Five imputations were regarded as sufficient as we were primarily interested in the point estimates [9].

We observed differential dropout in the RCT. Drop-out was particularly high in the active control group between baseline and three-month follow-up, mainly due to disagreement with group assignment. Dropouts were censored at the time of dropout. Baseline characteristics did not differ significantly between dropouts and completers in the active control group. To address potential dropout bias, inverse probability of censoring weights (IPCW) were applied [10, 11]. Dropout predictors were identified in each imputed dataset based on variables with a p-value <0.25 from a full regression model, fitted separately for the intervention and active control groups. Unified predictors selected across both groups were used in the IPCW models to ensure comparability. Predictor correlations were assessed using Pearson correlations for continuous variables and Cramer's V for categorical variables. IPCW were estimated using pooled logistic regression models for the probability of remaining observed at each time point. Models were fitted separately by treatment group, first including time point only and then including time point, unified predictors, and time-dependent covariates. Unstabilized and stabilized time-dependent weights were derived from the predicted probabilities of remaining uncensored; weights were not truncated. Participants who dropped out and could no longer contribute covariate information were assigned a weight of 0. The resulting weights were used to account for dropout within strata of confounders by upweighting uncensored observations.

Intervention effect and relapse probability

We calculated intervention effects and relapse probabilities over the weighted pseudo-population in all five imputed datasets for both groups separately (intervention/ active control). The intervention effect reflecting the probability of becoming reduced lonely after the NBSP-FiN intervention was calculated from the DJGLS data and depended on the cycle of the Markov state-transition model. Observations that were not lonely in the beginning according to the DJGLS scale cut-off point of three (intervention group n=17, active control group n=18), were excluded from the analyses [8, 12]. Although cutoff points for the DJGLS exist to differentiate between not lonely, moderately lonely, and strongly lonely, we determined a specific point change as a meaningful improvement. Based on expert consensus, the intervention was considered as effective if participants reduced their loneliness by two or more points on the DJGLS. This accounts for individual differences in the baseline loneliness score and acknowledges an improvement in those who would still be classified as lonely

according to the scale. We used the calculated DJGLS loneliness score at baseline, three, six and twelve months. Reduced loneliness was defined as an improvement (meaning reduction) of ≥2 points in the DJGLS score at three, six, and twelve months. Relapses were defined as a subsequent increase of ≥2 points in DJGLS score at six or twelve months among those who had previously improved at three or six months. For the first cycle, baseline to three-months trial data was used reflecting the direct intervention effect. The probability of moving to 'Reduced Loneliness' in the second cycle after the NBSP intervention was calculated from three to six-months data from the trial. Six to twelve-months data for the intervention effect was used for the third and fourth cycle. AC also had an effect on the feelings of loneliness after three months, after six, and after twelve months. After the maintenance phase (follow-up time) of one year, no intervention effect was applied, based on estimates from RECETAS experts. The probability of moving to 'Reduced Loneliness' in the fifth and following cycles after receiving NBSP or AC (after one year) was assumed to be zero. A relapse rate reflecting the probability to relapse from 'Reduced Loneliness' to 'Relapsed Loneliness' state, that can occur earliest after the second cycle, was calculated from the RECETAS trial data. All participants where the DJGLS increased again by two or more points, were considered in 'Relapsed Loneliness'. After one year, no relapse probability was applied, it was assumed to be zero for NBSP and AC. The categorization of the continuous loneliness scale was necessary as the Markov state transition model is based on discrete health states.

To quantify the effect of the intervention (risk ratios), we fitted a weighted generalized linear model (GLM) with a Poisson distribution and a log link. The log link models the logarithm of the probability of the outcome, allowing us to directly estimate risk ratios with 95% confidence intervals between the groups moving to 'Reduced Loneliness' [13]. A modified Poisson regression model was chosen as the preferred method over a log-binomial model to estimate risk ratios [14-16]. Predicted probabilities for each participant for each imputation for reduced loneliness were calculated from the GLMs where fitted coefficients reflect the weighting in the model. Weighted contingency tables for each of the five imputations of intervention/ active control group and reduced loneliness outcome were generated, and proportions of participants with reduced loneliness were calculated within each group to have the effect of becoming reduced loneliness in each group. All results were pooled over the five imputed datasets using Rubin's rule [17]. The same approach was performed for 'Relapsed Loneliness'. To evaluate the relapse to loneliness again, we fitted weighted GLMs with IPCW comparing participants in the NBSP intervention group with those in the active control group. From

the weighted models, relative risks with 95% confidence intervals of the five imputations were estimated. Predicted probabilities for each participant for each imputation for relapsed lonely were calculated from the regression models where fitted coefficients reflect the weighting in the model. The absolute relapse probability observed in AC was used as a baseline, and a relative risk multiplier was applied to estimate relapse in NBSP [18]. Intervention and relapse probability between six and twelve months were recalculated to a three-month probability to match the cycle length.

In a scenario analysis, we adjusted for baseline imbalances. Sex and flourishing score were the only two variables that were significantly different between the intervention and control group at baseline ($p<0.05$).

Health-related quality of life for 'Loneliness' and 'Reduced Loneliness'

To derive EQ-5D-5L utility values for 'Loneliness' and 'Reduced Loneliness', we restricted participants to those classified as lonely at baseline. For 'Loneliness', we restricted the dataset to lonely participants at three, six, or twelve months and fitted a weighted linear regression model over each imputed dataset with EQ-5D utility value as the dependent variable and IPCW weight statements to calculate a weighted utility for lonely over all time points. For 'Reduced Loneliness', we restricted for participants classified as reduced lonely and fitted a weighted linear regression model over each imputed dataset with EQ-5D utility value as the dependent variable and IPCW weight statements to calculate a weighted utility for being reduced loneliness over all time points.

All statistical analyses were performed in RStudio. Additional details on the statistical procedures are available from the corresponding author upon reasonable request.

Decision-analytic model

Health states

The following 18 health states were considered in the model:

- 'Loneliness & Well', 'Loneliness & Depression/Anxiety', 'Loneliness & T2DM', Loneliness & Dementia, ' 'Loneliness & CVD',
- 'Reduced Loneliness & Well', 'Reduced Loneliness & Depression/Anxiety', 'Reduced Loneliness & T2DM', 'Reduced Loneliness & Dementia', 'Reduced Loneliness & CVD',
- 'Relapsed Loneliness & Well', 'Relapsed Loneliness & Depression/Anxiety', 'Relapsed Loneliness & T2DM', 'Relapsed Loneliness & Dementia', 'Relapsed Loneliness & CVD',
- 'Death', 'Loneliness-/ disease-related Death' and 'Death by Suicide'.

Probability for developing loneliness-related diseases

To estimate transition probabilities of developing each included disease, disease-specific incidence rates were searched for the Spanish population and assumed to be valid for reduced loneliness people. Spanish age- and gender-specific incidence rates of the diseases from the Institute for Health Metrics and Evaluation (IHME) (GBD 2021) [19] were included. Incidence in the IHME database is defined as "The number of new cases of a given cause during a given period in a specified population. It is often approximated by taking the number of new cases in a year divided by the mid-year population size." [20]. According to the definition, this is the incidence proportion and can be used as a probability to develop the disease. The probability to develop the disease was recalculated for a cycle length of three months and adjusted with the gender distribution of the trial's participants.

To determine the impact of loneliness on the included diseases, effect size parameters (relative risk, hazard ratios, or odds ratios) comparing not lonely with lonely individuals were implemented in the model. To calculate the probability to develop a disease if lonely, the respective probability was transformed to a rate, multiplied by the relative risk / hazard ratio / odds ratio, and transformed back to a probability. We assumed that the odds ratio approximates a relative risk since the outcome (depression) is rare (<10%) in the non-exposed group [21, 22]. We assumed that the probability of developing the loneliness-related diseases are the same for both well individuals and individuals suffering from another loneliness-related disease.

Mortality

Spanish all-cause mortality and cause-specific mortality rates by age- and gender for CVD, Alzheimer's disease and other dementias, and T2DM were derived from IHME database [19]. Cause-specific mortality data was not available for depressive and anxiety disorders as these conditions do not directly cause death by the definition of the IHME. Instead, they increase the risk of death, which is ultimately attributed to an underlying physical cause.

Cause-specific mortality rates, defined as "The number of deaths due to cause divided by the mid-year population" [20], were transformed into a three-month probability. For the included diseases, mortality rates by causes, sex and age (deaths per 100,000 inhabitants for each age group) were derived from the IHME database. Mortality rates are defined based on the chapters of the WHO International Statistical Classification of Diseases and Related Health Problems 10th Revision (ICD-10) (https://icd.who.int/browse10/2019/en#/G30). We calculated the age- and gender-specific case fatality rate (CFR), as the disease-specific mortality rate per diseased person, by dividing disease-

specific deaths per 100,000 inhabitants by the disease prevalence per 100,000. The CFR is higher than the cause-specific mortality rate per 100,000 inhabitants as it determines the mortality risk per year among people with the disease. The CFR was applied to die due to the disease in the disease states. For Depression & Anxiety, no cause-specific mortality rates exist. For depression and anxiety, as the mortality rate of Mental disorders (F24, F50.0-F50.5) goes only until the age of 50, no CFR could be calculated. Instead, a relative risk for mortality by depression of 1.71 (95% CI, 1.54–1.90) and mortality by anxiety of 1.43 (95% CI, 1.24–1.64) regardless of geographic location [23], were pooled using fixed-effect inverse-variance method to a RR of 1.60 (95% CI, 1.47-1.74).

To avoid double-counting deaths in disease states [18], we corrected the all-cause mortality in all health states for the disease-specific mortality and death by suicide, except for depression and anxiety. The background mortality was not corrected for Depression & Anxiety as people don't die directly from these conditions, they only have an increased risk to die, and the death is assigned the underlying physical cause of death. This corrected all-cause mortality was applied to individuals in all 'Reduced Loneliness', both well or diseased, states, dying not due to a modeled disease, suicide or loneliness.

For 'Loneliness' with a disease, we determined the impact of loneliness and a disease on mortality, by summing up the excess mortality rate of loneliness with the CFR for the disease. For depression and anxiety, the excess mortality rate if lonely was multiplied with the combined relative risk for mortality by depression and anxiety.

Age and gender-specific suicide rates were retrieved from the Spanish National Statistics Institute (INE) [24]. Additionally, we considered the impact of loneliness on mortality and on suicide as well as the effect of diseases on suicide. We assumed that odds ratios approximate a relative risk if the outcome in the non-exposed group is rare (<10%) [21, 22]. A meta-analysis reporting an odds ratio of 1.26 (95% CI, 1.04-1.53) reflecting the increased mortality from loneliness was included [25]. The excess mortality for lonely individuals was calculated by multiplying the all-cause mortality rate by the mortality risk factor associated with loneliness followed by subtracting the all-cause mortality. For all 'Loneliness' states, independent of well or diseased, the all-cause mortality was additionally corrected for the excess mortality of loneliness. For 55 years old and above, an odds ratio of 3.48 (95% CI, 2.98–4.07) for suicide was reported for living alone or suffering from loneliness, which was used as it fits best to the age of our population [26]. A pooled increased hazard ratio of 1.58 (95% CI, 1.25-1.99) for suicide death due to anxiety and depression [27] and an odds ratio of 1.44 (95% CI, 1.08–1.93) due to CVD was integrated in our model [28]. A meta-analysis reported an odds ratio of

1.30 (95% CI, 0.81-2.10) between dementia and the risk of suicide ideation [29]. We assumed that the odds ratio approximates a relative risk since the outcome (suicide) is rare (<10%) in the non-exposed group [21, 22]. A meta-analysis showed a relative risk of 1.65 (95% CI, 0.95–2.85) for diabetes (T2DM) and increased risk of suicide [30]. Although the odds ratios were not significant, they were used in the model with its uncertainty interval, as there is a low likelihood to be one and to estimate the most likely number in the model.

Decision-analytic model validation

Face validation of the conceptual decision-analytic model, input parameters, assumptions and transition pathways was performed in several meetings with experts in decision modeling and RECETAS trial collaborators with expertise in the field of loneliness and its consequences on health and wellbeing. Internal validation of the model was performed by an independent researcher. Therefore, the TECH-VER checklist was assessed and all input parameters, formulas, links and calculations were checked for consistency and correctness. The TECH-VER, AdViSHE and CHEERS checklist can be provided upon request.

For external validation, we compared model outcomes with independent published results: Turning all loneliness parameters off, not correcting the background mortality for all diseases, using females/males gender only, using a cycle length of 1 month, changing the incidence of the diseases to 0, and switching half-cycle correction off, results in 30.63 remaining life years for females and 24.47 for males. This undiscounted life expectancy in our model is higher than the national statistics remaining life expectancy for a 61-year old female person of 26.57 years and 22.08 years for a 61-year old male person in 2021 [31]. However, the gap can be explained through the assumption of a healthy start population in our model. No diseases are prevalent in the beginning in both strategies, leading to overestimation of the remaining life expectancy. But as we aimed to evaluate the difference between NBSP and AC, this can be neglected.

References

1. EuroQol Research Foundation, *EQ-5D-5L User Guide*. 2019.
2. de Jong-Gierveld, J. and van Tilburg, T.G., *Manual of the Loneliness Scale. Methoden en technieken.* 1999.
3. Brick, J.M. and Montaquila, J.M., *Nonresponse and Weighting*, in *Handbook of Statistics - Sample Surveys: Design, Methods and Applications*. 2009. p. 163-185.
4. Haziza, D., *Imputation and Inference in the Presence of Missing Data*, in *Handbook of Statistics - Sample Surveys: Design, Methods and Applications*. 2009. p. 215-246.
5. Si, Y., et al., *Nonresponse Bias Analysis in Longitudinal Studies: A Comparative Review with an Application to the Early Childhood Longitudinal Study.* International Statistical Review, 2024. **92**(3): p. 383-405.
6. Peytchev, A., *Multiple Imputation for Unit Nonresponse and Measurement Error.* Public Opinion Quarterly, 2012. **76**(2): p. 214-237.
7. van Buuren, S. and Groothuis-Oudshoorn, K., *mice: Multivariate Imputation by Chained Equations in R.* Journal of Statistical Software, 2011. **45**(3): p. 1 - 67.
8. de Jong-Gierveld, J. and van Tilburg, T.G. *Manual of the Loneliness Scale (Updated from the printed version: 24-2-2022)*. 2022; Available from: https://home.fsw.vu.nl/tg.van.tilburg/manual_loneliness_scale_1999.html.
9. Stef van Buuren, *Flexible Imputation of Missing Data. 2.8 How many imputations? Available from: https://stefvanbuuren.name/fimd/sec-howmany.html*. 2018.
10. Cole, S.R. and Hernan, M.A., *Constructing inverse probability weights for marginal structural models.* Am J Epidemiol, 2008. **168**(6): p. 656-64.
11. Hernán, M.A. and Robins, J.M., *Per-Protocol Analyses of Pragmatic Trials.* N Engl J Med, 2017. **377**(14): p. 1391-1398.
12. de Jong-Gierveld, J. and van Tilburg, T.G. *Cut-off points for the De Jong Gierveld loneliness scale*. 1999; Available from: https://home.fsw.vu.nl/tg.van.tilburg/Cut-off%20points%20for%20the%20De%20Jong%20Gierveld%20loneliness%20scale.pdf.
13. Muller, C.J. and MacLehose, R.F., *Estimating predicted probabilities from logistic regression: different methods correspond to different target populations.* Int J Epidemiol, 2014. **43**(3): p. 962-70.
14. Chen, W., et al., *Comparing performance between log-binomial and robust Poisson regression models for estimating risk ratios under model misspecification.* BMC Med Res Methodol, 2018. **18**(1): p. 63.
15. Zou, G., *A modified poisson regression approach to prospective studies with binary data.* Am J Epidemiol, 2004. **159**(7): p. 702-6.
16. Gallis, J.A. and Turner, E.L., *Relative Measures of Association for Binary Outcomes: Challenges and Recommendations for the Global Health Researcher.* Ann Glob Health, 2019. **85**(1): p. 137.
17. Rubin, D.B., *Multiple Imputation for Nonresponse in Surveys*. 1987: Wiley.
18. Weinstein, M.C., et al., *Principles of good practice for decision analytic modeling in health-care evaluation: report of the ISPOR Task Force on Good Research Practices--Modeling Studies.* Value Health, 2003. **6**(1): p. 9-17.
19. Global Burden of Disease Collaborative Network. *Global Burden of Disease Study 2021 (GBD 2021) Results*. 2022; Available from: https://vizhub.healthdata.org/gbd-results/ [Access Date: 07.08.2024].
20. Institute for Health Metrics and Evaluation (IHME), *GBD 2021 Data and Tools Overview*. 2024, Institute for Health Metrics and Evaluation: Seattle, WA.

21. Zhang, J. and Yu, K.F., *What's the Relative Risk?: A Method of Correcting the Odds Ratio in Cohort Studies of Common Outcomes.* JAMA : the journal of the American Medical Association, 1998. **280**(19): p. 1690-1691.
22. Gidwani, R. and Russell, L.B., *Estimating Transition Probabilities from Published Evidence: A Tutorial for Decision Modelers.* Pharmacoeconomics, 2020. **38**(11): p. 1153-1164.
23. Walker, E.R., McGee, R.E., and Druss, B.G., *Mortality in mental disorders and global disease burden implications: a systematic review and meta-analysis.* JAMA Psychiatry, 2015. **72**(4): p. 334-41.
24. Instituto Nacional de Estadística (National Statistics Institute), *https://www.ine.es/en/ [Access Date: 30.01.2025]*. 2026.
25. Holt-Lunstad, J., et al., *Loneliness and Social Isolation as Risk Factors for Mortality: A Meta-Analytic Review.* Perspectives on Psychological Science, 2015. **10**(2): p. 227-237.
26. Alothman, D., et al., *The association between living alone, loneliness and suicide mortality and effect modification by age: A case:control study.* J Affect Disord, 2024. **352**: p. 278-280.
27. Li, X., et al., *Predictors of suicidal ideation, suicide attempt and suicide death among people with major depressive disorder: A systematic review and meta-analysis of cohort studies.* J Affect Disord, 2022. **302**: p. 332-351.
28. Chen, C., et al., *Patients with cardiovascular disorders and suicidality: A systematic review and meta-analysis.* Asian Journal of Psychiatry, 2023. **90**.
29. Desai, R., et al., *Suicide and dementia: A systematic review and meta-analysis of prevalence and risk factors.* Ageing Res Rev, 2024. **100**: p. 102445.
30. Wang, B., et al., *MANAGEMENT OF ENDOCRINE DISEASE: Suicide risk in patients with diabetes: a systematic review and meta-analysis.* Eur J Endocrinol, 2017. **177**(4): p. R169-R181.
31. Instituto Nacional de Estadística (National Statistics Institute), *Mortality tables by year, sex, age and functions. Available from: https://www.ine.es/jaxiT3/Datos.htm?t=27153 [Access Date: 30.01.2025]*. 2023.
